# Pellino-1 confers chemoresistance in lung cancer cells by upregulating cIAP2 through Lys63-mediated polyubiquitination

**DOI:** 10.18632/oncotarget.9619

**Published:** 2016-05-26

**Authors:** Yoon Kyung Jeon, Chung Kwon Kim, Jaemoon Koh, Doo Hyun Chung, Geun-Hyoung Ha

**Affiliations:** ^1^ Department of Pathology, Seoul National University Hospital, Seoul National University College of Medicine, Seoul 03080, Republic of Korea; ^2^ Department of Molecular Cell Biology, Sungkyunkwan University School of Medicine, Suwon 16419, Gyeonggi-do, Republic of Korea; ^3^ Department of Biomedical Sciences, Seoul National University College of Medicine, Seoul 03080, Republic of Korea

**Keywords:** Pellino-1, lung cancer, E3 ligase, oncogene, cIAP2

## Abstract

Pellino-1 is an E3 ubiquitin ligase that mediates immune receptor signaling pathways. The role of Pellino-1 in oncogenesis of lung cancer was investigated in this study. Pellino-1 expression was increased in human lung cancer cell lines compared with non-neoplastic lung cell lines. Pellino-1 overexpression in human lung cancer cells, A549 and H1299 cells, increased the survival and colony forming ability. Pellino-1 overexpression in these cells also conferred resistance to cisplatin- or paclitaxel-induced apoptosis. In contrast, depletion of Pellino-1 decreased the survival of A549 and H1299 cells and sensitized these cells to cisplatin- and paclitaxel-induced apoptosis. Pellino-1 overexpression in A549 and H1299 cells upregulated the expression of inhibitor of apoptosis (IAP) proteins, including cIAP1 and cIAP2, while Pellino-1 depletion downregulated these molecules. Notably, Pellino-1 directly interacted with cIAP2 and stabilized cIAP2 through lysine63-mediated polyubiquitination via its E3 ligase activity. Pellino-1-mediated chemoresistance in lung cancer cells was dependent on the induction of cIAP2. Moreover, a strong positive correlation between Pellino-1 and the cIAP2 expression was observed in human lung adenocarcinoma tissues. Taken together, these results demonstrate that Pellino-1 contributes to lung oncogenesis through the overexpression of cIAP2 and promotion of cell survival and chemoresistance. Pellino-1 might be a novel oncogene and potential therapeutic target in lung cancer.

## INTRODUCTION

Pellino family proteins (Pellino-1, 2, and 3) are E3 ubiquitin ligases that contains C-terminal RING-like domains [1]. Pellino proteins play an important role in Toll-like receptor (TLR), IL-1 receptor and T-cell receptor (TCR) signaling, by catalyzing Lys48- and Lys63-linked polyubiquitination of signaling molecules [2]. Briefly, Pellino-1 functions as a critical mediator for NF-κB activation in TLR3 and TLR4 signaling through its Lys63-linked ubiquitin ligase activity [3]. Pellino-1 is also implicated in MyD88-mediated TLR signaling and subsequent MAPK, ERK, and JNK activation [4, 5]. Moreover, Pellino-1 positively regulates type I interferon responses in human bronchial epithelial cells after TLR3 stimulation or rhinovirus infection [6]. In contrast, Pellino-1 acts as a negative regulator of TCR signaling by promoting Lys48-linked polyubiquitination and proteasomal degradation of REL, an NF-κB subunit [7]. Meanwhile, the expression of Pellino-1 increases following the TLR or TCR signaling [8].

We previously employed transgenic mice to assess the gain of function for Pellino-1. We demonstrated for the first time that Pellino-1 functions as a novel oncogene, contributing to B-cell lymphomagenesis through BCL6 stabilization via Lys63-polyubiquitination [9]. However, little is known concerning the oncogenic role of Pellino-1 in solid tumors. Pellino-1-transgenic mice showed macroscopic and microscopic tumors of adenocarcinoma histology in the lungs. Thus, we hypothesized that Pellino-1 might be involved in the oncogenesis of lung adenocarcinoma.

Lung cancer is the leading cause of cancer-related death worldwide [10]. Recent advances in molecular targeted therapy have improved the clinical outcome of patients with adenocarcinoma [11]. However, conventional cytotoxic chemotherapy still plays a key role in the management of lung cancer patients with no druggable target [12]. Thus, the identification of resistance mechanisms to cytotoxic chemotherapy are needed.

The inhibitor of apoptosis (IAP) proteins block apoptosis by directly inhibiting caspases, thereby contributing to chemoresistance of cancer cells [13]. In addition, several IAPs such as XIAP, cIAP1, and cIAP2 have E3 ligase activity and activate NF-κB and MAPK signaling pathway [14]. Thus, cIAPs also promote tumor cell proliferation, invasion, and metastasis [15]. IAPs are considered promising therapeutic targets, and IAP antagonists are under clinical trials for cancer treatment [16]. Lung cancers show overexpression of XIAP, cIAP1, and cIAP2, although their association with responsiveness to chemotherapy has been conflicting [17, 18]. Amplification of genetic loci for cIAP1 and cIAP2 was observed in subset of lung cancers [19]. However, the mechanism regulating cIAPs expression in lung cancer remains elusive.

In the present study, we showed that Pellino-1 expression is elevated in human lung cancer cells and increases cell proliferation and survival. Notably, Pellino-1 was found to directly interact and stabilize cIAP2 through Lys63-polyubiquitination, thus promoting chemoresistance in lung cancer cells.

## RESULTS

### Pellino-1 is variably expressed in lung cancer cell lines and associated with toll-like receptors (TLRs) expression

Pellino-1 and TLR expression pattern was evaluated in lung cancer cell lines and non-neoplastic lung cell lines. Overall, Pellino-1 expression was increased in lung cancer cell lines compared with non-neoplastic cells (Supplementary Figure S1A). Pellino-1 expression tended to be correlated with TLR expressions, particularly with TLR3, TLR5, TLR7, and TLR9 expression (Supplementary Figure S1A). When lung cancer cell lines were treated with TLR agonists, Pellino-1 expression was usually up-regulated depending on types of cell lines and TLR agonists (Supplementary Figure S1B and S1C). In addition, stimulation of A549 cells with TLR agonist for TLR3 (poly(I:C)), TLR4 (LPS), or TLR8 (ssRNA40) increased survival fraction (Supplementary Figure S1D). These data suggested that TLRs signaling might induce Pellino-1 expression in lung cancer cell lines. To investigate the role of Pellino-1 in lung cancer, Pellino-1 relatively high expressing A549 cells and Pellino-1 moderately expressing H1299 cells were used throughout this study.

### Pellino-1 promotes cell survival in lung cancer cells

**Figure 1:**
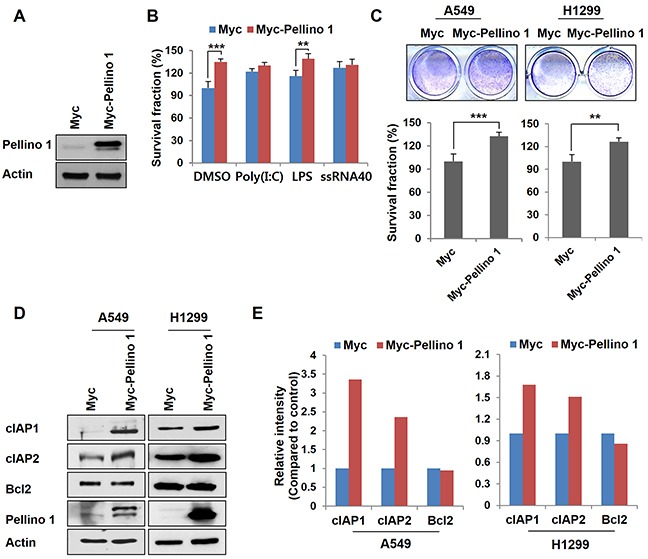
Overexpression of Pellino-1 increases the survival of lung cancer cells **A and B.** A549 cells were transfected with Myc or Myc-tagged Pellino-1 (Myc-Pellino-1) expression plasmids, then subjected to immunoblotting with anti-Pellino-1 and anti-actin antibodies (A). Pellino-1-overexpressing A549 cells were unstimulated (DMSO) or stimulated with Poly (I:C), LPS, or ssRNA40. At 7 days after stimulation, cells were incubated with crystal violet coloration, and the intensity of crystal violet was determined by spectrometry at 570 nm. The percentage of surviving fractions was determined by the intensity of crystal violet relative to Myc-transfected and DMSO-treated cells (B). **C.** A549 and H1299 cells were transfected with Myc or Myc-Pellino-1. At 7 days after transfection, cells were incubated with crystal violet coloration, and the intensity of crystal violet was determined. The percentage of surviving fractions was determined by the intensity of crystal violet relative to Myc-transfected cells. **D and E.** Cell lysates of Pellino-1-overexpressing A549 and H1299 cells were analyzed by immunoblotting with anti-cIAP1, anti-cIAP2, anti-Bcl2, anti-Pellino-1, and anti-actin antibodies (D). The expression of the indicated proteins was quantified using densitometry. Histograms represent the intensity of protein normalized to actin (E). The results are representative of three independent experiments. All *P* values were calculated using unpaired Student's t test. **P < 0.01; ***P < 0.005.

Overexpression of Pellino-1 in A549 and H1299 cells using Myc-Pellino-1 transfection led to a significant increase in the survival rate (Figure 1A–1C). A Pellino-1-mediated increase in cell survival was also observed in A549 cells stimulated with the TLR4 ligand (LPS) (Figure 1B). In addition, overexpression of Pellino-1 markedly increased the expression of cIAP1 and cIAP2 but not that of Bcl2 protein in A549 and H1299 cells (Figure 1D and 1E).

Because Pellino-1 activates NF-κB activation in immune cells [20, 21], the effect of Pellino-1 on NF-κB activation was examined in BEAS-2B (non-neoplastic bronchial epithelial cells) and A549 cells. Pellino-1 overexpression activated NF-κB pathways in these cells as shown by phospho-p65 and Rel-B upregulation and increased nuclear translocation of NF-κB subunits (Supplementary Figure S2). Together, these data suggest that Pellino-1 might promote cell survival through the upregulation of cIAPs and NF-κB activation in lung cancer cells.

### Pellino-1 promotes chemoresistance in lung cancer cells and Pellino-1 knockdown increases the chemosensitivity of lung cancer cells

**Figure 2:**
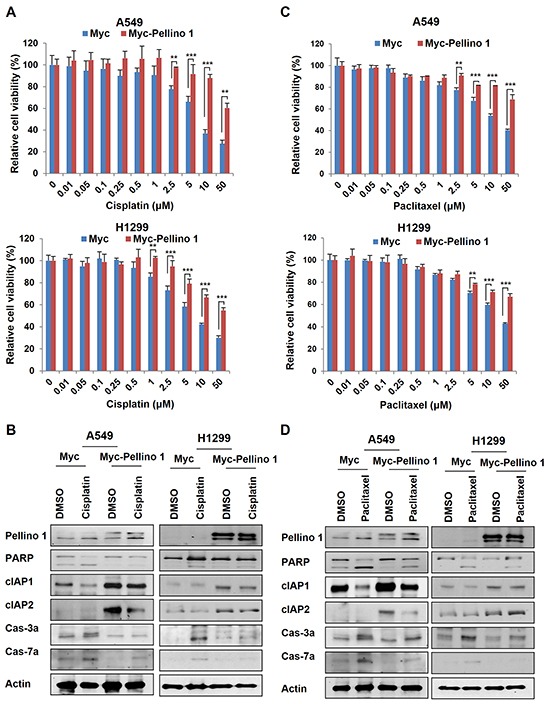
Pellino-1 overexpression promotes the chemoresistance of lung cancer cells **A.** Pellino-1-overexpressing A549 and H1299 cells were cultured in 96-well plates (200 μl cell suspensions, 2 × 10^4^ cells/ml) and treated with cisplatin at variable concentrations. At 72 hours after treatment, the MTT assay was performed to estimate the cell viability. Data represent the mean ± SD of at least three independent experiments. **B.** A549 or H1299 cells were transfected with Myc or Myc-Pellino-1 expression plasmids. At 36 hours after transfection, cells were treated with 5 μM cisplatin for 24 hours. Cells were harvested and then subjected to immunoblotting with anti-Pellino-1, anti-PARP, anti-cIAP1, anti-cIAP2, anti-cleaved caspase-3 (Cas-3a), anti-cleaved caspase-7 (Cas-7a), and anti-actin antibodies. **C.** Pellino-1-overexpressing A549 and H1299 cells were cultured in 96-well plates (200 μl cell suspensions, 2 × 10^4^ cells/ml) and treated with paclitaxel at variable concentrations. At 72 hours after treatment, the MTT assay was performed. Data represent the mean ± SD of at least three independent experiments. **D.** A549 or H1299 cells were transfected with Myc or Myc-Pellino-1. At 36 hours after transfection, cells were treated with 5 μM paclitaxel for 24 hours. Cells were harvested and then subjected to immunoblotting with indicated antibodies. All *P* values were calculated using unpaired Student's t test. **P < 0.01; ***P < 0.005.

Since Pellino-1 overexpression upregulated cIAP1 and cIAP2 expression and activated NF-κB pathway, we hypothesized that Pellino-1 would be implicated in the responsiveness to chemotherapy in lung cancer cells. A549 and H1299 cells with Pellino-1 overexpression showed chemoresistance to cisplatin and increased cell viability than control cells (Figure 2A and Supplementary Figure S3A). Cisplatin-induced cleavage of caspase-3, caspase-7, and PARP (activities suggestive of apoptosis) was consistently decreased in A549 and H1299 cells with Pellino-1 overexpression compared with that in control cells, which showed more proteolytic cleavage of caspase-3, caspase-7 and PARP following cisplatin treatment (Figure 2B). A similar result was observed when Pellino-1-overexpressed A549 and H1299 cells were treated with paclitaxel (Figure 2C and 2D; Supplementary Figure S3B).

**Figure 3:**
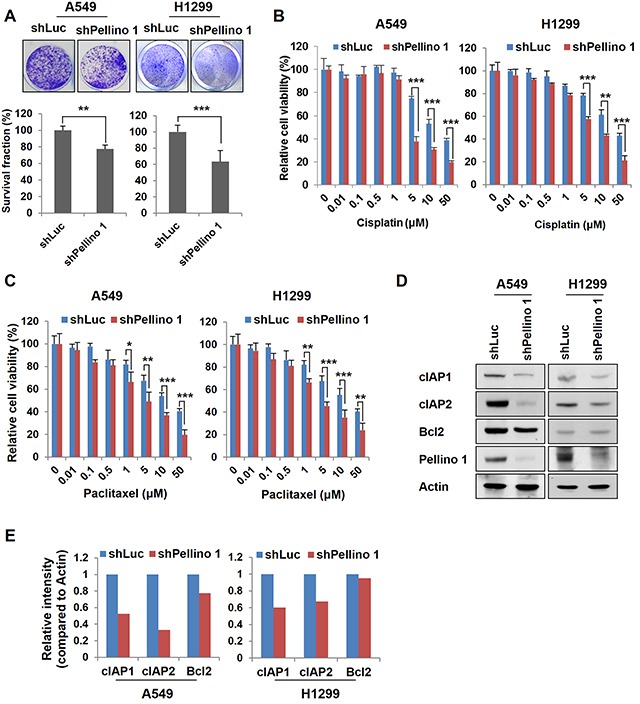
Depletion of Pellino-1 leads to the chemosensitization of lung cancer cells **A.** A549 and H1299 cells were transfected with control luciferase shRNA (shLuc) or Pellino-1-targeted shRNA (shPellino-1) expression plasmids. At 7 days after transfection, cells were incubated with crystal violet coloration, and the intensity was determined by spectrometry at 570 nm. The percentage of surviving fractions was determined by the intensity of crystal violet relative to shLuc-transfected cells. **B and C.** A549 and H1299 cells were transfected with shLuc or shPellino-1 and treated with cisplatin (B) or paclitaxel (C) at variable concentrations. At 72 hours after treatment, the MTT assay was performed. Data represent the mean ± SD of at least three independent experiments. **D and E.** Cell lysates from A549 and H1299 cells with Pellino-1 depletion were analyzed by immunoblotting with anti-cIAP1, anti-cIAP2, anti-Bcl2, anti-Pellino-1, and anti-actin antibodies (D). Expression of indicated proteins was quantified using densitometry. Histograms represent the intensity of indicated protein normalized to actin (E). The results are representative of three independent experiments. All *P* values were calculated using unpaired Student's t test. *P < 0.05; **P < 0.01; ***P < 0.005.

Furthermore, knockdown of Pellino-1 using shPellino-1 in A549 and H1299 cells reduced the cell survival compared with control cells (Figure 3A) and sensitized these cells to cisplatin or paclitaxel (Figure 3B and 3C). Of note, Pellino-1-knockdown reduced cIAP1 and cIAP2 expression (Figure 3D and 3E).

These data suggest that Pellino-1 overexpression promotes resistance to chemotherapeutic agent-induced apoptosis in A549 and H1299 cells putatively via the upregulation of cIAP1 and cIAP2 expression.

### Pellino-1 interacts with cIAP2, and its binding promotes the stability of cIAP2 via Lys63-mediatied polyubiquitination

**Figure 4:**
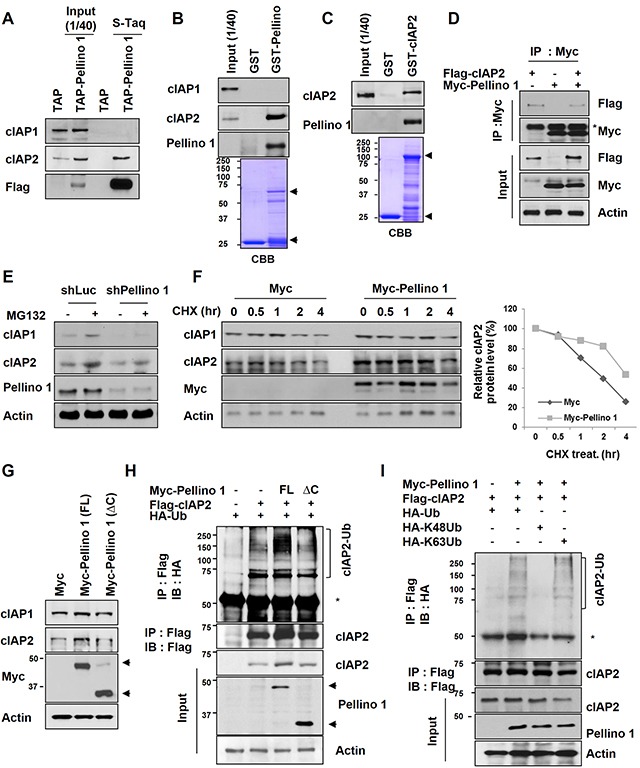
Pellino-1 interacts with cIAP2 and induces cIAP2 stabilization via K63-mediated polyubiquitination **A.** Cell lysates from 293T and 293T cells stably expressing Flag-Pellino-1 were isolated by tandem affinity purification (TAP). Bound proteins were immunoblotted with anti-cIAP1, anti-cIAP2, and anti-Flag antibodies. **B.** A549 cell lysates were incubated with GST or GST-Pellino-1, and bound proteins were subjected to immunoblotting with indicated antibodies. **C.** A549 cell lysates were incubated with GST or GST-cIAP2, and bound proteins was subjected to immunoblotting with indicated antibodies. **D.** HCT116 cells were transfected with Myc, Myc-Pellino-1, and/or Flag-cIAP2 plasmids. At 48 hours after transfection, the cells were harvested, and 2 mg of total cellular lysate were subjected to immunoprecipitation (IP) using anti-Myc antibody followed by analysis by immunoblotting using anti-Flag, anti-Myc, or anti-actin antibodies. **E.** A549 cells transfected with shLuc or shPellino-1 were treated with the proteasome inhibitor MG132 (10 μM). Six hours later, cells were harvested and subjected to immunoblotting with anti-cIAP1, anti-cIAP2, anti-Pellino-1, and anti-actin antibodies. **F.** A549 cells were transfected with Myc or Myc-Pellino-1. At 36 hours after transfection, cells were treated with cycloheximide (CHX) for indicated times and subjected to immunoblotting with anti-cIAP1, anti-cIAP2, anti-Myc, and anti-actin antibodies (*left*). The level of cIAP2 protein expression was quantified by densitometry and normalized with actin as the internal control (*right*). **G.** A549 cells were transfected with Myc, Myc-Pellino-1-full length (FL), or Myc-Pellino-1-ΔC (RING domain deletion). At 36 hours after transfection, cells were subjected to immunoblotting with anti-cIAP1, anti-cIAP2, anti-Myc, and anti-actin antibodies. **H.** HCT116 cells were transfected with Myc-Pellino-1-FL or Myc-Pellino-1-ΔC in combination with HA-Ub and Flag-cIAP2 plasmids. At 36 hours after transfection, cells were harvested for immunoprecipitation with an anti-Flag antibody and then subjected to immunoblotting with anti-HA and anti-Flag antibodies. **I.** HCT116 cells were transfected with Myc-Pellino-1, Flag-cIAP2, HA-Ub, HA-K48Ub, and/or HA-K63Ub in combination. At 36 hours after transfection, cells were harvested for immunoprecipitation with an anti-Flag antibody and then subjected to immunoblotting with anti-HA, anti-Flag, anti-Myc, or anti-actin antibodies.

Because expression of Pellino-1, an E3 ligase, was positively correlated with cIAP1 and cIAP2 protein expression, we investigated whether Pellino-1 directly interacts with cIAP1 and cIAP2 and regulates their stabilities. Extracts from asynchronously growing 293T cells stably expressing TAP-tagged Pellino-1 were subjected to pull-down assays (Figure 4A). In addition, GST-Pellino-1 fusion proteins were incubated with cellular extracts from asynchronized A549 cells (Figure 4B), and GST-cIAP2 fusion proteins were incubated with purified His-tagged Pellino-1 (Figure 4C). These pull-down assays together revealed that Pellino-1 directly interacts with cIAP2 protein but not cIAP1 (Figure 4A–4C). Interaction of Pellino-1 and cIAP2 proteins were also demonstrated in HCT116 cells ectopically expressing these proteins (Figure 4D).

The mechanism by which Pellino-1 upregulates cIAP2 protein expression was explored using a Pellino-1 knockdown or overexpression system. Treatment with a proteasome inhibitor (MG132) increased the cIAP2 level in both shLuc- and shPellino-1-transfected A549 cells, indicating that cIAP2 protein stability might be regulated by Pellino-1 via a proteasome pathway (Figure 4E). Consistently, the degradation of cIAP2 protein was markedly delayed in Pellino-1-overexpressing A549 cells treated with cycloheximide (CHX, inhibitor of protein biosynthesis) compared with control cells (Figure 4F). Overexpression of full-length Pellino-1 (Pellino-1-FL) increased the cIAP2 protein level, whereas the Pellino-1 C-terminal RING domain deletion mutant (Pellino-1-ΔC), which lacks E3 ligase activity, had little effect on the cIAP2 level in A549 cells (Figure 4G). The ubiquitination assay showed that the polyubiquitinated form of cIAP2 was consistently increased in HCT116 cells transfected with Pellino-1-FL, but not in cells transfected with Pellino-1-ΔC (Figure 4H). Finally, the ubiquitination assay using HCT116 cells transfected with Myc-Pellino-1 in combination with HA-Ub, HA-K48Ub, HA-K63Ub, and/or Flag-cIAP2 revealed that Pellino-1 overexpression increased the amount of total and the K63 polyubiquitinated form of cIAP2, but not the K48 polyubiquitinated form (Figure 4I). Taken together, these data indicate that Pellino-1 directly interacts with cIAP2, thereby promoting the stabilization of cIAP2 through Lys63-mediatied polyubiquitination.

### Stabilization and overexpression of cIAP2 account for the chemoresistance induced by Pellino-1

**Figure 5:**
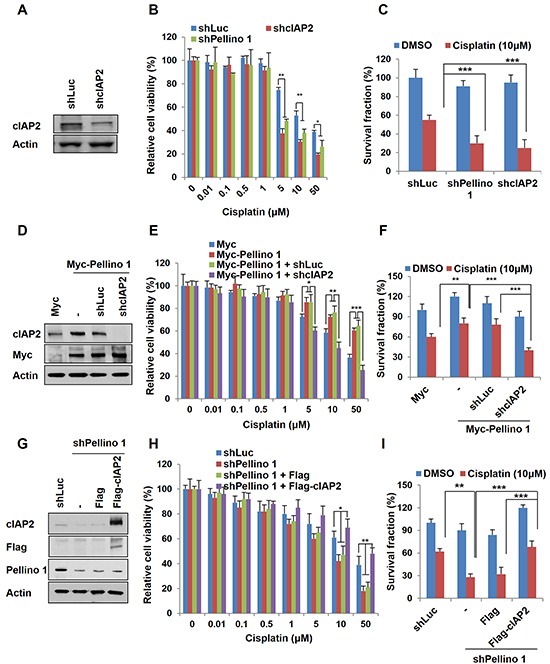
Pellino-1 leads to the chemoresistance of lung cancer via cIAP2 stabilization **A.** A549 cells were transfected with shLuc or shcIAP2 plasmids. At 48 hours after transfection, cells were harvested and subjected to immunoblotting with anti-cIAP2 and anti-actin antibodies. **B.** A549 cells were transfected with shLuc, shcIAP2, or shPellino-1. After 24 hours of transfection, cells were treated with cisplatin, and cell viability was measured by the MTT assay. **C.** A549 cells were transfected with shLuc, shcIAP2, or shPellino-1. At 24 hours after transfection, cells were treated with cisplatin for 5 days and then incubated with crystal violet to determine the survival fraction. **D.** Pellino-1-overexpressing A549 cells were transfected with shLuc or shcIAP2 plasmids. At 48 hours after transfection, cells were harvested and subjected to immunoblotting with anti-cIAP2, anti-Myc, and anti-actin antibodies. **E.** Pellino-1-overexpressing A549 cells were transfected with shLuc or shcIAP2. After 24 hours of transfection, cells were treated with cisplatin, and cell viability was measured by the MTT assay. **F.** Pellino-1-overexpressing A549 cells were transfected with shLuc or shcIAP2. At 24 hours after transfection, cells were treated with cisplatin for 5 days and then incubated with crystal violet to determine the survival fraction. **G.** Pellino-1-knockdown A549 cells were transfected with Flag or Flag-cIAP2. At 48 hours after transfection, cells were harvested and subjected to immunoblotting with anti-cIAP2, anti-Flag, anti-Pellino-1, and anti-actin antibodies. **H.** Pellino-1-knockdown A549 cells were transfected with Flag or Flag-cIAP2. After 24 hours of transfection, cells were treated with cisplatin, and cell viability was measured by the MTT assay. **I.** Pellino-1-knockdown A549 cells were transfected with Flag or Flag-cIAP2. At 24 hours after transient transfection, cells were treated with cisplatin for 5 days and then incubated with crystal violet to determine the survival fraction. All *P* values were calculated using the unpaired Student's t test. *P < 0.05; **P < 0.01; ***P < 0.005.

We next investigated whether cIAP2 contributes to Pellino-1-induced chemoresistance. Depletion of cIAP2 reduced the cell viability and survival fraction in A549 cells treated with cisplatin at similar levels observed in Pellino-1-depleted A549 cells (Figure 5A–5C). Moreover, cIAP2 knockdown counteracted the chemoresistant effect of Pellino-1 overexpression and reduced the cell viability and survival in A549 cells treated with cisplatin (Figure 5D–5F). In contrast, cIAP2 overexpression recovered the cell viability and survival in Pellino-1-depleted A549 cells treated with cisplatin (Figure 5G–5I). These data indicate that cIAP2 might play an important role in the Pellino-1-induced cell survival and chemoresistance.

### Pellino-1 expression is positively correlated with cIAP2 expression in human lung adenocarcinoma

**Figure 6:**
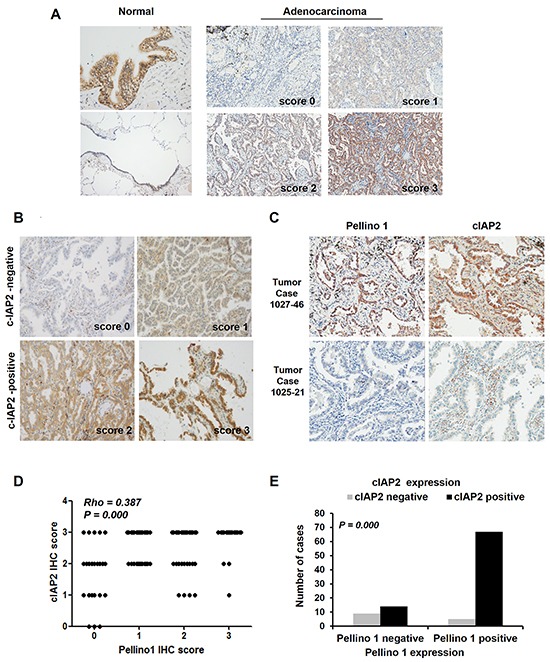
Correlation between Pellino-1 and cIAP2 expression in human lung adenocarcinomas **A.** Pellino-1 protein expression was analyzed in human normal lung tissue (left) and lung adenocarcinoma tissues (right) by immunohistochemistry (IHC), and representative images are shown. **B.** Representative images of IHC staining for cIAP2 with variable IHC score in lung adenocarcinoma tissues are shown (original magnification, ×400). **C.** Representative IHC images for Pellino-1 and cIAP2 in patients who showed high expression for both proteins (upper) or who showed little expression for both proteins (lower) (original magnification, ×400). **D.** The scatter plot represents the correlation between the Pellino-1 and cIAP2 IHC score in 95 patients with lung adenocarcinoma. Spearman *r* and *P* value are indicated. **E.** The association between Pellino-1 and cIAP2 expression was also analyzed by the Pearson χ^2^ test after dichotomizing patients into cIAP2 negative (*n* = 14) and cIAP2 positive (*n* = 81) groups.

Finally, the association between Pellino-1 and cIAP2 expression was assessed using immunohistochemistry (IHC) in 95 patients with lung adenocarcinoma. Pellino-1 was highly expressed in bronchial epithelial cells and moderately to weakly expressed in alveolar macrophages and pneumocytes (Figure 6A, *left*). Pellino-1 and cIAP2 were expressed by human lung adenocarcinoma tissues with variable intensities (Figure 6A and 6B). Representative IHC images for Pellino-1 and cIAP2 from patients with lung adenocarcinoma who showed strong expression of both Pellino-1 and cIAP2 or who showed little expression of both proteins are shown in Figure 6C. The Pellino-1 and cIAP2 expression levels were scored in four grades by IHC. Briefly, a significantly positive correlation between Pellino-1 and cIAP2 IHC scores was observed (Spearman's *Rho* = 0.387, *P* = 0.000) (Figure 6D). Moreover, patients with Pellino-1 expression (IHC score 1-3) showed significantly higher cIAP2 expression (IHC score 2-3) (*P* = 0.000 by Pearson χ^2^ test) (Figure 6E).

## DISCUSSION

The present study demonstrates that Pellino-1 promotes cell survival and chemoresistance by directly binding and stabilizing cIAP2 protein via Lys63-polyubiquitination in lung cancer cells. This represents a novel mechanism by which Pellino-1 contributes to oncogenesis.

cIAPs play an important role in cancer cell survival by antagonizing apoptosis [22]. cIAP1 and cIAP2 also function as critical molecules in the activation of NF-κB pathways upon TNF receptor or TLR stimulation via their E3 ligase activities [22, 23]. By doing this, cIAPs influence a multitude of cellular processes to drive cell survival, chemoresistance, and cell migration [13, 15, 22, 24, 25]. Overexpression of cIAPs was associated with chemoresistance and the poor prognosis of patients in multiple cancer types, including lung cancer [17, 18, 22, 26]. In contrast, IAP antagonists, including mitochondrial SMAC, are released upon apoptotic stimuli and interact with IAPs to inhibit their functions [22]. Thus, the inhibition of IAPs using SMAC mimetic emerged as a promising therapeutic strategy in cancers including lung cancer [27]. However, lung cancer cells developed resistance to SMAC mimetic by upregulating cIAP2 through NF-κB and PI3K pathways [28]. Thus, an understanding of the regulatory mechanism of cIAPs is important for the control of cancer.

cIAP expression is known to be regulated at the transcriptional, translational, and post-translational levels [29–31]. This study for the first time demonstrated that cIAP2 is directly stabilized through Pellino-1-mediated Lys63-polyubiquitination. Of note, it was previously reported that Pellino-1 mediates the TRAF6-induced Lys63-polyubiquitination and stabilization of cIAP2 in microglial cells in the central nervous system upon TLR signaling. cIAP2 subsequently promotes the Lys48-polyubiquitination and degradation of TRAF3 (a negative regulator of MAPK). Consequently, Pellino-1 contributes to MAPK activation and proinflammatory cytokine production and neuroinflammation [5, 8]. However, whether cIAP2 is a direct target of Pellino-1 E3 ligase remains unknown. We have herein clearly shown that Pellino-1 directly binds to cIAP2 and functions as a direct E3 ligase for cIAP2 through in vitro ubiquitination and pull-down assays. Moreover, a significant positive correlation between Pellino-1 and cIAP2 expression was observed in human lung adenocarcinoma tissues. Pellino-1 overexpression promoted cell proliferation and conferred resistance to cisplatin and paclitaxel in lung cancer cells, which was dependent on cIAP2. These results suggest that the Pellino-1/cIAP2 axis might be a potential therapeutic target in lung cancer.

This study has limitations in that only two cell lines were evaluated for the oncogenic role of Pellino-1 and the correlation between Pellino-1 and cIAP2 in human lung cancer tissues was moderate (rho value of 0.4). The latter observation suggested that cIAP2 level might also be regulated by mechanisms other than Pellino-1. In terms of Pellino-1 as a potential therapeutic target in lung cancer, Pellino-1 expression tended to be higher in lung cancer cell lines compared to non-neoplastic cell lines. In addition, Pellino-1 expression was strong, moderate and weak by IHC in 18% (17/95), 31% (29/95) and 27% (26/95) of human lung adenocarcinoma tissues, respectively. A lung adenocarcinoma genome data from The Cancer Genome Atlas (TCGA) showed Pellino-1 mRNA upregulation or gene amplification in 7% of patients and The Human Protein Atlas displayed Pellino-1 expression in 10 of 12 cases with non-small cell lung cancer. Thus we think that high Pellino-1 expression level would provide a potential biomarker for future targeting of Pellino-1 in lung cancer. In addition, given that chemosensitizing effect by Pellino-1 depletion was relatively modest in lung cancer cells in this study, Pellino-1 targeting might be beneficial when combined with other chemotherapeutic strategies used in the treatment of lung cancer.

In this study, cIAP1 did not directly bind to Pellino-1, although cIAP1 expression increased by Pellino-1 overexpression. Pellino-1 activated NF-κB in lung cancer cells. Based on these observations and previous reports [32], it is hypothesized that cIAP1 might be upregulated by NF-κB signaling activated by Pellino-1, a finding that should be proved by further study. We previously reported that Pellino-1 contributes to B-cell lymphomagenesis through BCL6 stabilization [9]. Although little has been known for the oncogenic role of BCL6 in epithelial malignancies, a recent study reported that microRNA-187-3p inhibited lung cancer development by targeting oncogenic BCL6 [33]. Thus, there is a possibility that Pellino-1 might contribute to oncogenesis of lung cancer through BCL6 stabilization, which remains clarified by further study.

Pellino-1 was discovered as an E3 ligase that catalyzes TLR signaling molecules [8]. Mounting evidence has suggested that TLR signaling might be implicated in oncogenesis [34], including in lung cancer [35–37]. In lung cancer, TLR7 and TLR8 signaling promoted cell proliferation and chemoresistance and was associated with the poor survival of patients [35, 38]. In addition, TRAF6, an important molecule in the TLR/IL-1R pathway, is overexpressed in lung cancer tissues and related to a chemoresistance [39]. In this study, TLR stimulation in lung cancer cells increased Pellino-1 expression, indicating that TLR signaling and Pellino-1 might be cooperatively involved in the oncogenesis of lung cancer. The TLR agonists for TLR3 (poly(I:C)) and TLR8 (ssRNA40) increased the survival of A549 cells but with no additive effect by Pellino-1 overexpression, whereas LPS (TLR4 agonist) increased the survival of A549 cells at a higher level together with Pellino-1 overexpression. These data suggest that Pellino-1 might function as an important downstream molecule in TLR4-mediated oncogenesis and chemoresistance in lung cancer cells. However, further studies are needed to understand the connection between Pellino-1 and TLRs in lung cancer development and progression.

In summary, this study has shown that Pellino-1, an E3 ligase, contributes to the promotion of cell survival and chemoresistance through cIAP2 upregulation by Lys63-polyubiquitination and subsequent stabilization in lung cancer. Pellino-1 is suggested to be a novel oncogene that plays an important role in lung oncogenesis and might be a potential therapeutic target.

## MATERIALS AND METHODS

### Cell culture, stable cell lines, and cell isolation

The fourteen human lung cell lines (A549, Calu-3, Calu-6, H322, H358, H441, H460, H1299, H1264, HCC1833, H1838, and H1975 [all adenocarcinoma except for H460, large cell carcinoma], BEAS-2B [non-neoplastic bronchial epithelial cells], WI-26 [lung fibroblastic cells]), and 293T cells were purchased from the American Type Culture Collection (Manassas, VA, USA). HCT116 cells were kindly gifted from Bert Vogelstein (Johns Hopkins Kimmel Cancer Center, Baltimore, MD, USA). These cell lines were grown in Bronchial Epithelial Cell Growth Medium (BEGM), RPMI-1640, DMEM or McCoy's Medium (Hyclone, Logan, UT, USA), 10% FBS contained with 1% penicillin G/streptomycin in a humidified 5% CO_2_ atmosphere.

A cell line stably overexpressing tandem affinity purification (TAP)-Pellino-1 was generated by transfecting TAP-Pellino-1 or the empty vector TAP (as a control) into HEK293T cells.

### Plasmid construction and transfection

The full-length cDNA sequence of human Pellino-1 was amplified using oligodT primers. Pellino-1-ΔC includes the N-terminal 280 amino acids and lacks the C-terminal RING domain. Pellino-1-FL was subcloned into Myc-, GST-, or TAP-tagged fusion plasmids. The pEGFP-N3 vector encoding TAP (Strep-Flag-tagged) was kindly provided by Dr. Hong Tae Kim (Sungkyunkwan University, Suwon, Korea). cIAP2 was subcloned into pcDNA3.0-Flag- and pGEX-KG-6P (GST-tagged) fusion plasmids. The Myc-cIAP2 construct was kindly provided by Dr. Soo Young Lee (Ewha Womans University, Seoul, Korea). For shRNA synthesis, the following gene-specific sequences were generated using the pSuper vector (Oligoengine, Seattle, WA, USA): the Pellino-1-targeted sh RNA (shPellino-1), 5′-GGGTTCAACACACTAGCAT-3′; the human cIAP2 shRNA (shcIAP2), 5′-CAGTTCGTACATTT CTTTCAT-3′; and the luciferase shRNA (shLuc, as a control), 5′-CTACGCGGAATACTTCGA-3′. For transient transfection, cells were electroporated using a microporator (Digital Biotechnology, Seoul, Korea) according to the manufacturer's instructions.

### Antibodies and reagents

The following antibodies were used for subsequent studies: anti-Pellino-1 (F-7), anti-Bcl2, anti-cIAP1, anti-cIAP2, anti-TRAF6 (Santa Cruz Biotechnology, Santa Cruz, CA, USA), anti-TLR1, anti-TLR2, anti-TLR3, anti-TLR4, anti-TLR5, anti-TLR7, anti-TLR8, and anti-TLR9 (IMGENEX, San Diego, CA, USA), anti-actin, anti-Flag M2 (Sigma, St. Louis, MO, USA), anti-p-p65, anti-p65, anti-Rel-B, anti-p52, anti-p50, anti-TAK1, anti-PARP, anti-caspase 3 active (Cas-3a), anti-caspase 7 active (Cas-7a) (Cell signaling Technology, Danvers, MA, USA), anti-RIP (BD Biosciences, San Diego, CA, USA), anti-Lamin B1 (Abcam, Cambridge, UK), anti-HA, and anti-Myc (Roche, Basel, Switzerland) antibodies.

The following reagents were used for TLRs agonists: 5 mg/ml Poly(I:C) (TLR3 agonist), 5 mg/ml LPS (TLR4 agonist), and 5 mg/ml ssRNA40 (TLR8 agonist) (InvivoGen, San Diego, CA, USA). Cisplatin and paclitaxel (Selleck Chemicals, Houston, TX, USA), 25 μM MG132, 100 μg/ml CHX, dimethyl sulfoxide (DMSO) (A.G. Scientific, San Diego, CA, USA), and 3-(4,5-dimethylthiazol-2-yl)-2,5-diphenyltetrazolium bromide (MTT) (Sigma, St. Louis, MO, USA) were purchased.

### Cell viability and survival clonogenic assays

Cell viability and proliferation were measured using the MTT assay. Briefly, cells were harvested from exponential-phase cultures and plated in 96-well flat-bottomed microliter plates and then treated with cisplatin or paclitaxel. After the indicated time points, the cells were incubated with 2 mg/ml MTT substrate for 3 hours and then solubilized with DMSO. The optical density was spectrophotometrically measured at 490 nm.

For survival clonogenic assays, A549 or H1299 cells with Pellino-1 overexpression or depletion were seeded at various densities in 12-well plates and treated with cisplatin or paclitaxel. At 7 days after treatment, cells were incubated with 0.05% crystal violet coloration, and the intensity of crystal violet was spectrophotometrically determined at 570 nm. The surviving fractions was determined by the intensity of crystal violet. Survival curves were generated from an average of three independent experiments.

### Western blot analysis and cellular fractionation

For immunoblot analysis, the cells were harvested and lysed in nuclear extraction (NE) buffer [20 mM HEPES (pH 7.6), 20% glycerol, 250 mM NaCl, 1.5 mM MgCl_2_, 0.1% Triton X-100, 1 mM phenylmethylsulfonyl fluoride (PMSF), 1 mM dithiothreitol (DTT), and protease inhibitor cocktail (PIC) (Roche)]. Equal amounts of proteins were separated by SDS-PAGE and analyzed by immunoblotting with indicated antibodies.

For cellular fractionation, cytoplasmic fractions were prepared using PA buffer [150 mM Tris–HCl (pH 7.5), 150 mM NaCl, 1 mM EDTA, 1 mM PMSF, and 1 mM DTT, and PIC]. Nuclear fractions were collected by the dissolution of nuclei in XBE2 buffer [10 mM HEPES (pH 7.5), 300 mM NaCl, 1 mM EDTA, 2 mM MgCl_2_, 1 mM PMSF, 1 mM DTT, and PIC].

### In vitro binding and immunoprecipitation assays and in vivo ubiquitination assays

For the S-tag pull down assay, 293T cells adapted to suspension conditions were grown in DMEM supplemented with 10% FBS and 1% penicillin G/streptomycin. Cell pellets were lysed in NETN buffer [100 mM NaCl, 1 mM EDTA, 20 mM Tris-HCl (pH 8.0), 0.5% Nonidet P-40] containing with 1 mM PMSF, 1 mM DTT, and PIC. Cell lysates were cleared by centrifugation, and the supernatants were incubated with 20 μl of streptavidin-Sepharose beads (Amersham Biosciences, Piscataway, NJ, USA) for 8 hours at 4°C. After washing the beads, the bound proteins were separated by 8% SDS-PAGE and analyzed by immunoblotting.

For the GST pull-down assay, the fusion proteins were absorbed onto glutathione-protein A/G Sepharose beads (Amersham Biosciences) and incubated with whole-cell extracts from A549 cells. The bound proteins were separated by 8% SDS-PAGE and analyzed by immunoblotting.

For immunoprecipitation, HCT116 cells were transfected with Myc, Myc-Pellino-1, or Flag-cIAP2 plasmids. Forty-eight hours after transfection, the cells were harvested and lysed in immunoprecipitation buffer [50 mM Tris-HCl (pH 7.5), 150 mM NaCl, 1% NP40, 1 mM EDTA, 1 mM PMSF, 1 mM DTT, and PIC]. Each cell extract (2 mg of total protein) was incubated with antibodies against Myc for 2 hours at 4°C followed by incubation with protein-A/G Sepharose beads for an additional 2 hours. The beads were pelleted, washed, and analyzed by immunoblotting. For the in vivo ubiquitination assay, HCT116 cells were transfected with plasmids encoding Myc, Myc-tagged Pellino-1-FL, or Pellino-1-ΔC and HA-tagged ubiquitin (HA-Ub), HA-tagged ubiquitin K48 (HA-K48Ub), or HA-tagged ubiquitin K63 (HA-K63Ub) in combination. Thirty-six hours post-transfection, the cells were harvested, and the cell lysates from each plate were collected into two aliquots. One aliquot (10%) was used for conventional immunoblotting, and the other (90%) was used for immunoprecipitation with the anti-Flag antibody followed by immunoblotting.

### Immunohistochemistry for human lung cancer tissues

Formalin-fixed, paraffin-embedded tumor tissues from 95 patients with lung adenocarcinoma were collected, and a 2-mm-diameter tissue microarray was constructed for IHC. This study was approved by the Institutional Review Board of Seoul National University Hospital (H-1211-049-440). IHC was performed using the Benchmark XT automated staining system (Ventana Medical Systems, Tucson, AZ, USA) with antibodies to Pellino-1 (F-7) (Santa Cruz Biotechnology) and cIAP2 (AF817; R&D systems, Minneapolis, MN, USA) The expression patterns were evaluated based on the intensity and proportion of staining in tumor cells and were scored using the following scale: 0, negative; 1, weak; 2, moderate; or 3, strong staining intensity in >10% of tumor cells. Statistical analyses were performed using SPSS version 21 (IBM Corp., Armonk, NY, USA). Comparisons between variables were performed using the χ^2^ test or Spearman's correlation test, and two-sided *P* values of <0.05 were deemed statistically significant.

## SUPPLEMENTARY FIGURES


