# Supplementary material for: Pellino-1 confers chemoresistance in lung cancer cells by upregulating cIAP2 through Lys63-mediated polyubiquitination
Source: Oncotarget. 2016 May 26;7(27):41811–24. doi: 10.18632/oncotarget.9619 (PMC5173098; doi:10.18632/oncotarget.9619)
Supplement: Supplementary file 1 [file oncotarget-07-41811-s001.pdf]

Pellino-1 confers chemoresistance in lung cancer cells by upregulating cIAP2 through Lys63-mediated polyubiquitination

SUPPLEMENTARY FIGURES

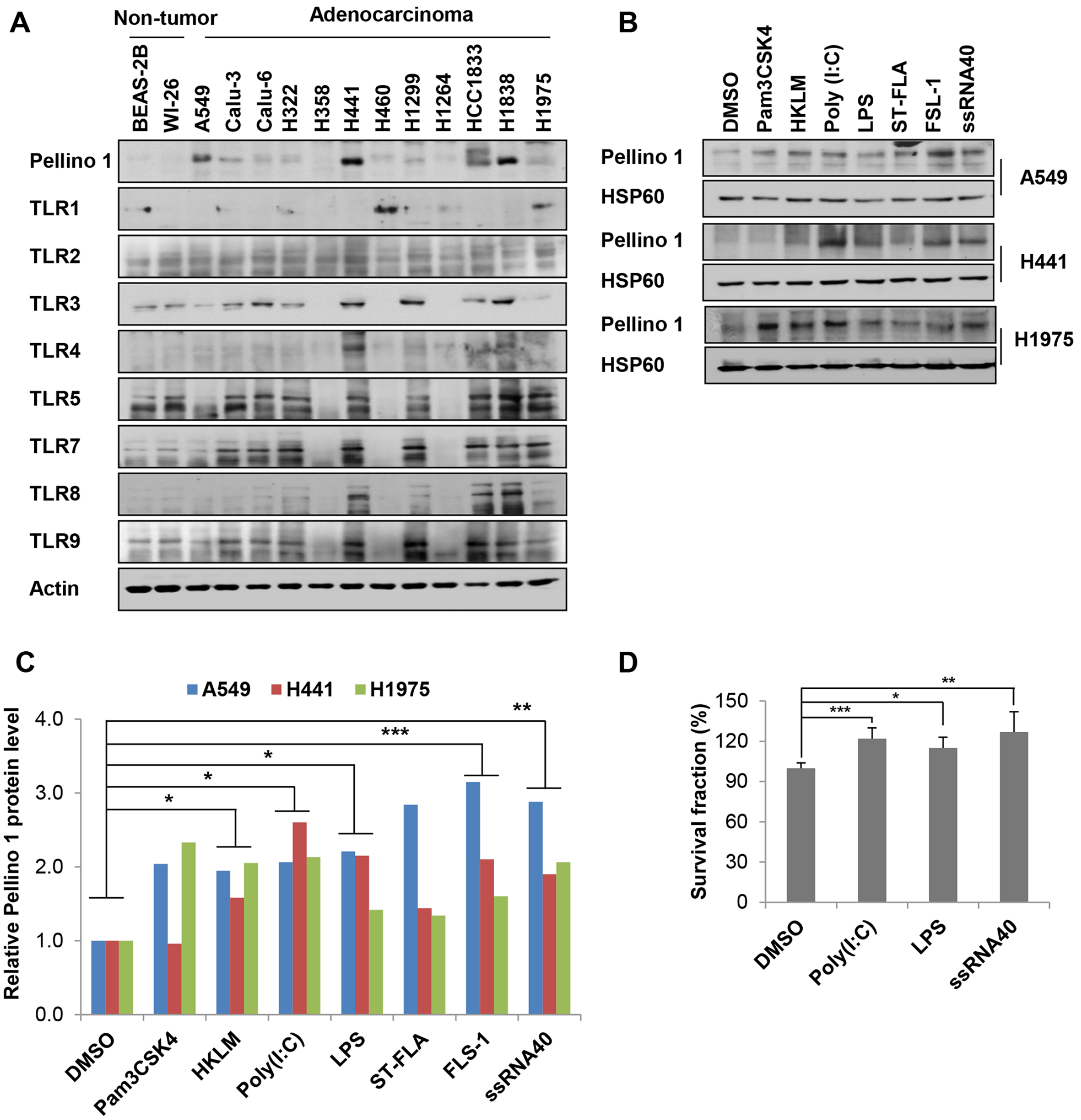

Supplementary Figure S1: The expression correlation between toll-like receptors (TLRs) and Pellino-1 in lung cancer cell lines. A. Two non-tumor lung cell lines (BEAS-2B [non-neoplastic bronchial epithelial cell lines] and WI-26 [lung fibroblastic cell lines]) and twelve lung cancer cell lines (A549, Calu-3, Calu-6, H322, H358, H441, H460, H1299, H1264, HCC1833, H1838, H1975; all adenocarcinoma except for H460, large cell carcinoma) were harvested and subjected to immunoblotting for Pellino-1 and indicated TLRs. **B and C.** A549, H441 and H1975 cells were treated with TLRs agonist against TRL1/2 (Pam3CSK4, 50 ng/ml), TLR2 (HKLM, 10^7 cells/ml), TLR3 (Poly (I:C), 5 ug/ml), TLR4 (lipopolysaccharide (LPS), 5 ug/ml), TLR5 (ST-FLA, 5 ug/ml), TLR6/2 (lipopeptide FSL-1 (FSL-1), 100 ng/ml) or TLR8 (ssRNA40, 5 ug/ml) for 36 hours. Cells were harvested and subjected to immunoblotting with anti-Pellino-1 and anti-HSP60 (loading control) antibodies (B). Pellino-1 protein expression was quantitated using Image J software as normalized to HSP60 level. Histograms represent relative Pellino-1 protein level in TLRs treated cells compared to DMSO treated cells. Results are represented of three independent experiments (C). **D.** A549 cells were stimulated with 5 ug/ml Poly (I:C), 5 ug/ml LPS, or 5 ug/ml ssRNA40 at days 0, 2, 4 and 6. At 7 days, cells incubated with crystal violet coloration. Intensity of crystal violet was then determined by spectrometry at 570 nm. DMSO was used as control. All P values were calculated using unpaired Student's t test. *,$P < 0.05$; **, $P < 0.01$; ***, $P < 0.005$.

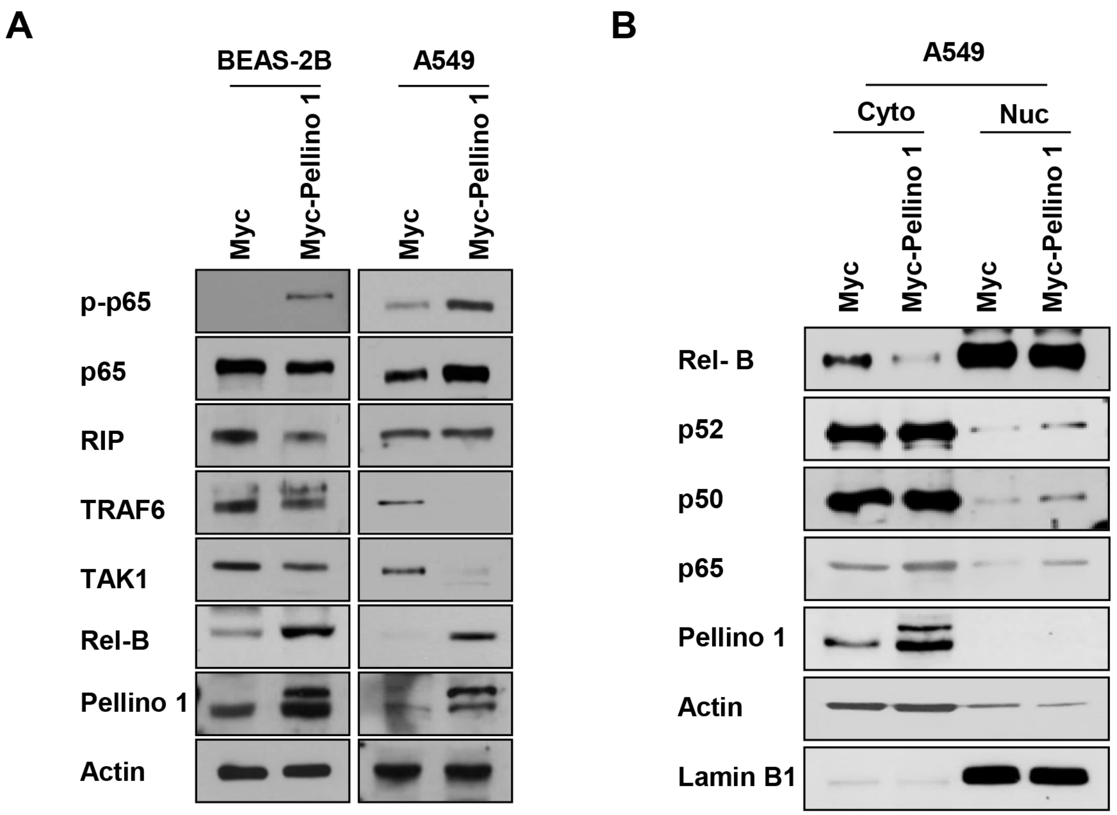

Supplementary Figure S2: The effects of NF-κB pathways by Pellino-1 overexpression in BEAS-2B and A549 cells. A. BEAS-2B or A549 cells were transfected with Myc or Myc-Pellino-1 expression plasmids. At 48 hours after transfection, cells were harvested and subjected to immunoblotting with indicates antibodies. **B.** A549 cells were transfected with Myc or Myc-Pellino-1 expression plasmids and harvested at 48 hours of transfection. The cell lysates were obtained separately from cytoplasmic (Cyto) and nuclear (Nuc) fractions and subjected to immunoblotting with anti-Rel-B, anti-p52, anti-p50, anti-p65, anti-Pellino-1, and anti-actin (as control cytoplasmic fraction) or lamin B1 (as a control for nuclear fraction) antibodies.

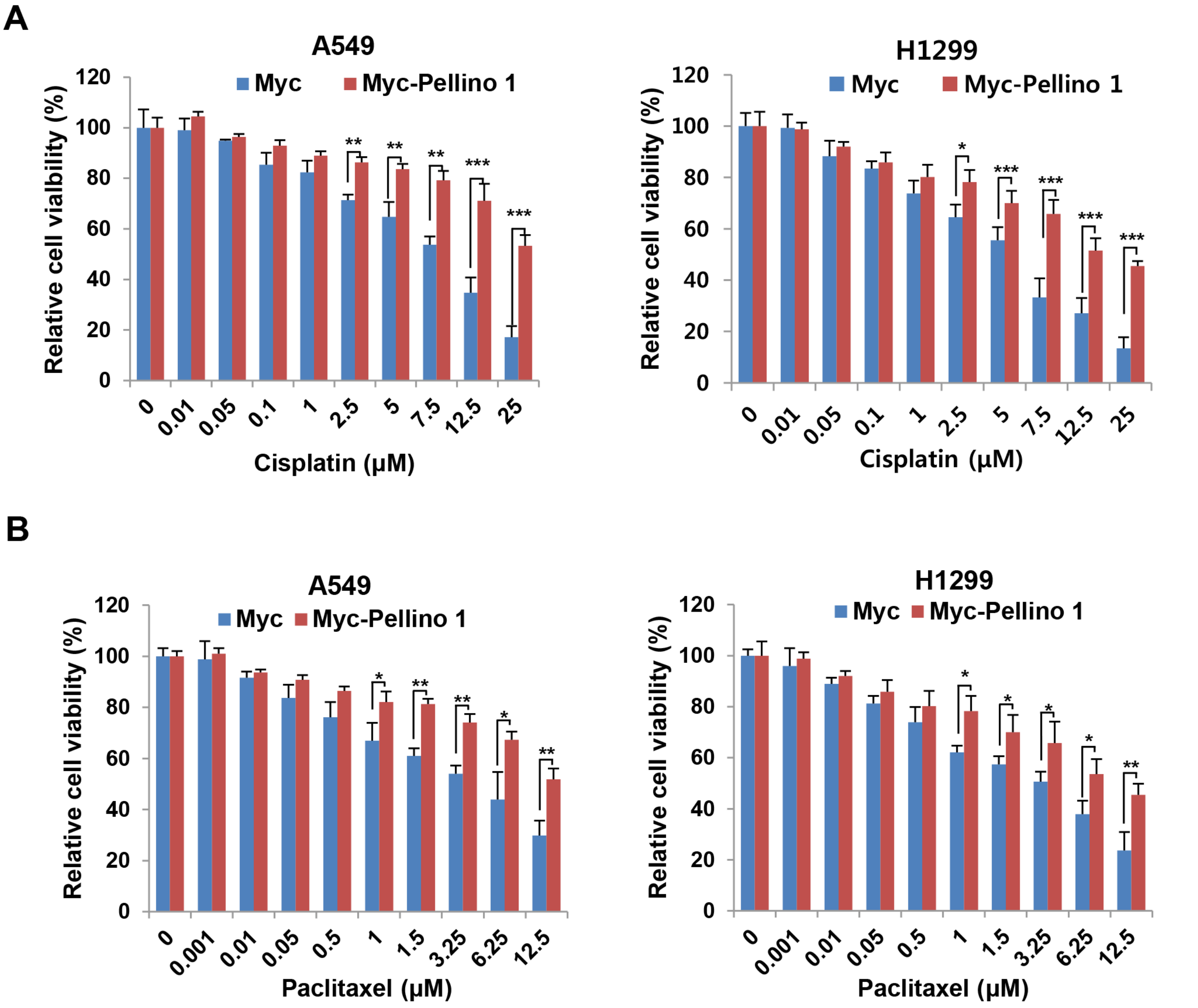

Supplementary Figure S3: Overexpression of Pellino-1 promotes the chemoresistance of lung cancer cells in A549 and H1299 cells. A. Pellino-1-overexpressing A549 and H1299 cells were cultured in 96-well plates (200 ml cell suspensions, 1×10^4 cells/ml) and treated with cisplatin at variable concentrations. At 72 hours after treatment, the MTT assay was performed to estimate the cell viability. Data represent the mean ± SD of at least three independent experiments. **B.** Pellino-1-overexpressing A549 and H1299 cells were cultured in 96-well plates (200 ml cell suspensions, 1×10^4 cells/ml) and treated with paclitaxel at variable concentrations. At 72 hours after treatment, the MTT assay was performed. Data represent the mean ± SD of at least three independent experiments. All *P* values were calculated using unpaired Student's t test. $^*P < 0.05$; $^{**}P < 0.01$; $^{***}P < 0.005$.
